# Supplementary material for: Comparative Transcriptome and sRNAome Analysis Suggest Coordinated Citrus Immune Responses against Huanglongbing Disease
Source: Plants (Basel). 2024 May 29;13(11):1496. doi: 10.3390/plants13111496 (PMC11175137; doi:10.3390/plants13111496)
Supplement: Supplementary file 1 [file plants-13-01496-s001.zip › supplementary_figures.pdf]

plants

MDPI

Supplementary Materials

Supplementary Materials and methods

1 *C*Las detection

24 weeks after *C*Las infection, *C*Las infected and mock samples from Shatangju and lemon were harvested. On the base of four different positions (east, west, north and south), two mature leaves from each position were taken from randomly selected plants. The leaves were washed and dehydrated, and veins surrounding tissues were excised. Samples were ground in mortar and pestle. After grinding, 200mg of ground sample was used for DNA extraction using a Plant DNeasy kit according to the manufacturer's directions (Axygen). Based on real-time PCR Ct values on the different concentration gradients of plasmids containing a fragment of *C*Las 16S rDNA, linear regression was calculated using *Ct* values. The equation Y = -0.286X + 11.924 represents the relationship between Y, the copy number of 16S rDNA, and X, which represents the Ct value of a specific real-time PCR reaction. The *C*Las titer, which represents the copy number of 16S rDNA in each sample, is determined using the formula T=100×10-0.286X+11.924, where T is the titer in copy/g FW. To determine *C*Las titer, a mixture was prepared with 250 nmol/L of primers, 150 nmol/L of probes, and 1 µl in each 20 µl real-time PCR system. The reactions were conducted on a LightCycler®480II (Roche) using the following cycles: Subject the sample to denaturation at a temperature of 95°C for 20 seconds, followed by a temperature of 95°C for 5 seconds and a temperature of 58°C for 40 seconds. Repeat this process for a total of 40 cycles.

2 Detection of Biochemical Components

2.1 Quantification of hydrogen peroxide (H_2O_2)

The level of H_2O_2 was assessed in both *C*Las infected lemon and Shatangju, as describe by [1]. Brifely, 200mg homogenized leaf powder with 3 mL of 5% trichloroacetic acid and 60mg of activated charcoal were used to extract H_2O_2. After centrifugation of the homogenate at 5000g for 20 minutes at 4°C, the supernatant was carefully collected. Subsequently, the absorbance of the supernatant was measured at a wavelength of 505 nm. The concentration of H_2O_2 was then determined utilizing a standard curve specific to H_2O_2 concentrations.

2.2 Determination of superoxide dismutase (SOD) activity

SOD activity was assessed using a modified method derived from [2]. In this method, 100 µL of a 20-fold diluted extract was combined with a solution containing 1 mM DETAPAC buffer (pH 7.8), 1.25 U catalase, 0.07 mM NBT, 0.2 mM xanthine, and 0.010 U xanthine oxidase, making a total volume of 1.0 mL. The activity of SOD was quantified based on its ability to inhibit the rate of NBT reduction at 560 nm and 25°C. One unit of SOD was defined as the quantity of enzyme necessary to induce a 50% inhibition of the NBT reduction rate under the specified conditions.

2.3 Determination of malondialdehyde (MDA) activity

The MDA content of leaves was determined using a thiobarbituric acid (TBA) reaction as described by [3]. For extraction, 100 mg of frozen leaf powder was homogenized with inert sand in 2.5 mL of 80% ethanol (v/v), followed by centrifugation at 3,000×g for 10 minutes at 4°C.

2.4 Determination of catalase (CAT) activity

Catalase (CAT) activity was determined following the method described by [4]. Briefly, the reaction mixture (1.1 mL) comprised 100 μL of crude enzyme extract, 37.8 mM sodium phosphate buffer (pH 7.0), and 4.4 mM H_2O_2. The reduction in absorbance was monitored at 240 nm. One unit of CAT activity was defined as the degradation of 1 micromole of H_2O_2 per minute at 25°C.

2.5 Determination of ascorbate peroxidase (APX) activity

Ascorbate peroxidase (APX) activity was assessed following a modified method outlined by [5]. The standard reaction mixture (1.0 mL) consisted of 0.17 mM of ascorbate, 33 μL of crude enzyme extract, and a 60.3 mM potassium phosphate buffer (pH 7.0). The reaction commenced upon addition of 4.95 mM H_2O_2. The rate of ascorbate oxidation was monitored at 290 nm over a period of 3 minutes. APX activity was quantified as the oxidation of 1 micromole of ascorbate per minute at 25°C.

2.6 Measurement of ion leakage

CLas infected lemon and Shatangju leaves were collected and cell death was assessed by measuring electrolyte leakage from the phloem exudates, which is indicated by an increase in conductivity. Following the collection of plant samples, the leaves were submerged in ultrapure water for a duration of 30 minutes. Subsequently, conductivity of the exudates extracted from phloem-enriched bark tissues was measured using a conductivity bench meter (INESA Scientific Instrument Co. Ltd， DDSJ-308F). Then, the samples were subjected to a brief autoclaving process in the identical solution, after which the conductivity was once again measured (referred to as total conductivity). The rise in electrolyte leakage (conductivity) is measured as a percentage of the overall amount. Experiment was repeated three times.

3 Starch grains and callose observation

24 weeks after inoculation with CLas, fresh symptomatic leaves of both lemon and Shatangju were collected for microscopic observation. Leaf veins of both citrus varieties were cut into small pieces approximately 1-2 mm in size and immediately immersed in a 2.5% glutaraldehyde + 4% paraformaldehyde solution prepared in a buffer system with a pH between 6.8 and 7.2 for fixation. The samples were fixed at 4°C for 3 hours and then embedded in Spurrresin. Tissues embedded in resin were sectioned using a Leica microtome (HistoCore AUTOCUT) with a thickness of 800 nm. To observe starch granules, sections were stained with 0.01% toluidine blue for 30 minutes and observed under a microscope (Zeiss AxioScope A1) in bright field. To detect callose, sections were stained with a 0.05% aniline blue solution for 10 minutes and observed under a microscope (Zeiss AxioScope A1) under UV light. Callose deposition was quantified by counting the number of fluorescent spots in the phloem of each sample [6].

3.1 Starch content quantification

The extraction and determination of starch content was conducted following the instructions outlined in the manual of the Plant Starch Detection Kit (Beijing Solabo Technology Co., Ltd., Beijing, China). 24 weeks after CLas infcetion, fresh symptomatic leaves of lemon and Shatangju were collected. Approximately 0.1g of midrib tissue from each citrus variety was weighed and then finely chopped into a grinding bowl with liquid nitrogen. The plant powder was mixed with 1ml of 80% ethanol and subjected to a 80°C water bath for 30 minutes. After centrifugation at 3000g for 5 minutes, the supernatant was decanted, and 0.5mL of double-distilled water was added to the residue, followed by heating in a boiling water bath for 15 minutes. Subsequently, 0.35mL of 60% perchloric acid was added to the

solution, which was then shaken on a benchtop shaker for 15 minutes at room temperature. After thorough mixing with 0.85mL of double-distilled water, the mixture was centrifuged at 3000g for 10 minutes to collect the supernatant for further analysis. Starch content was quantitatively determined using the sulfuric acid-anthrone colorimetric method [7].

4 Expressional Analysis of genes and miRNAs

4.1 RNA-seq validation by RT-qPCR

Gene-specific primer sets were used to perform quantitative real-time PCR analysis to validate the RNA-Seq analysis. 30 DEGs related to various biological processes such as cell wall metabolism, lipid metabolism, plant-pathogen interaction, protein phosphorylation and antioxidant activity were chosen. All primer sequences are listed in Supplementary Table S12. The procedures for plant culture, sample collection, and RNA extraction are outlined in the previous section. The process of cDNA synthesis was conducted for three distinct biological replicates. The complementary DNA (cDNA) was synthesized using the Superscript III First–Strand synthesis kit (Invitrogen, California, USA) according to the instructions provided by the manufacturer. The RT-qPCR analysis was conducted using the CFX Connect Real-Time System from (Bio-Rad Hercules, CA). The PCR mixture (20 mL) comprised 10 mL of TransStart® Green qPCR SuperMix from (TransGen Biotech, Beijing). 0.4 mL of each forward and reverse primer (10 mM), 1 mL cDNA template, and 8 mL ddH2O. For qPCR analysis, three technical replicates were performed for each biological replicate. The PCR process involved incubation at 95°C for 30 seconds, followed by 40 cycles of amplification at 95°C for 10 seconds and 60°C for 30 seconds, with fluorescence signal capture at the end of each 60°C step. The gene expression levels were determined using the $2^{-\Delta\Delta Ct}$ method [8]. The $\log_2$ for each sample was calculated as a ratio of the absolute expression value for the *C*Las infected plant to the mock-grafted control. Each gene log2 from RT-PCR was compared to the RNA-Seq analysis of the corresponding sample.

4.2 The expression analysis of small RNAs and their target genes

Two small RNAs, csi-miR399 and csi-miR166, reverse transcription primers and detection primers for the internal reference miR159, were designed using Primer Premier 5.0 software. Furthermore, detection primers for fluorescence quantitative PCR of small RNA target genes were also designed using Primer Premier 5.0 software. Fluorescence quantitative PCR detection was performed using the primers listed in Supplementary Table S12. The TAKARA SYBR Premix Ex TaqTM II reagent kit detected small RNA and its target genes. In a PCR tube on ice, 10 μL of SYBR Premix Ex TaqTM II Buffer, 0.4 μL each of primers F/R (10 μmol·L^-1), 7.2 μL of ddH2O, and 2 μL of template (cDNA) were added, with a total volume of 20 μL. The reaction program was set as follows: 95°C for 3 minutes, followed by 45 cycles of 95°C for 7 seconds, 57°C for 10 seconds, and 72°C for 15 seconds; finally, a melting curve analysis was performed. The experiment was conducted using a LightCycler 480 II real-time fluorescence quantitative PCR instrument (Roche).

Supplementary Figures

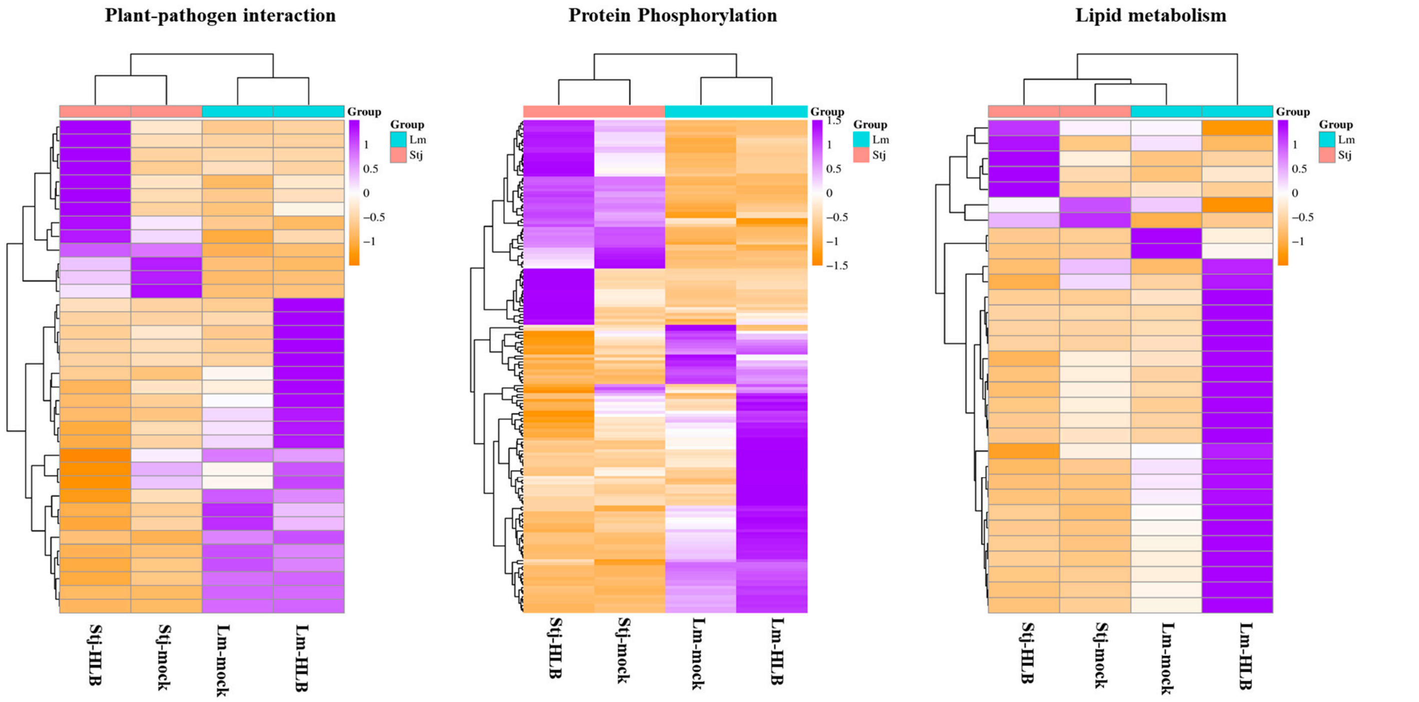

Figure S1: Comparative analysis of Shatangju and lemon DEGs. Expression profiling of DEGs between lemon and Shatangju involved in plant-pathogen interaction, protein phosphorylation and lipid metabolism. Purple color showes up regulation of DEGs while yellow showes down regulation. Stj= Shatangju, Lm= Lemon

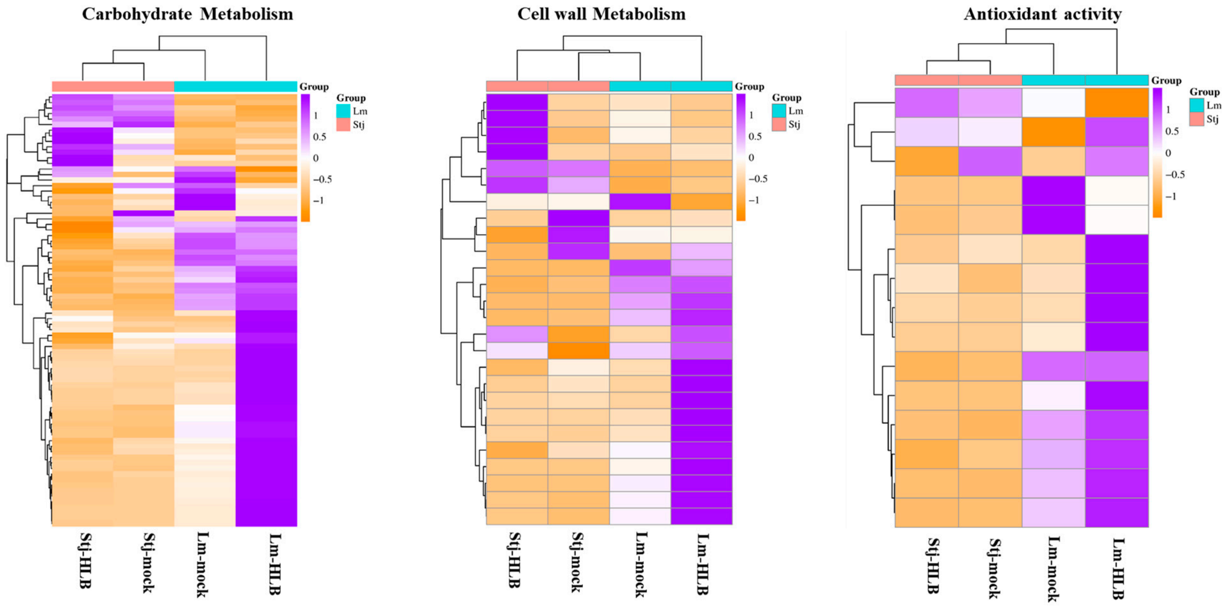

Figure S2: Comparative analysis of Shatangju and lemon DEGs. Heat maps of DEGs between lemon and Shatangju involved in carbohydrate metabolism, cell wall metabolism, and antioxidant activity. Purple color showes up regulation of DEGs while yellow showes down regulation. Stj= Shatangju, Lm= Lemon.

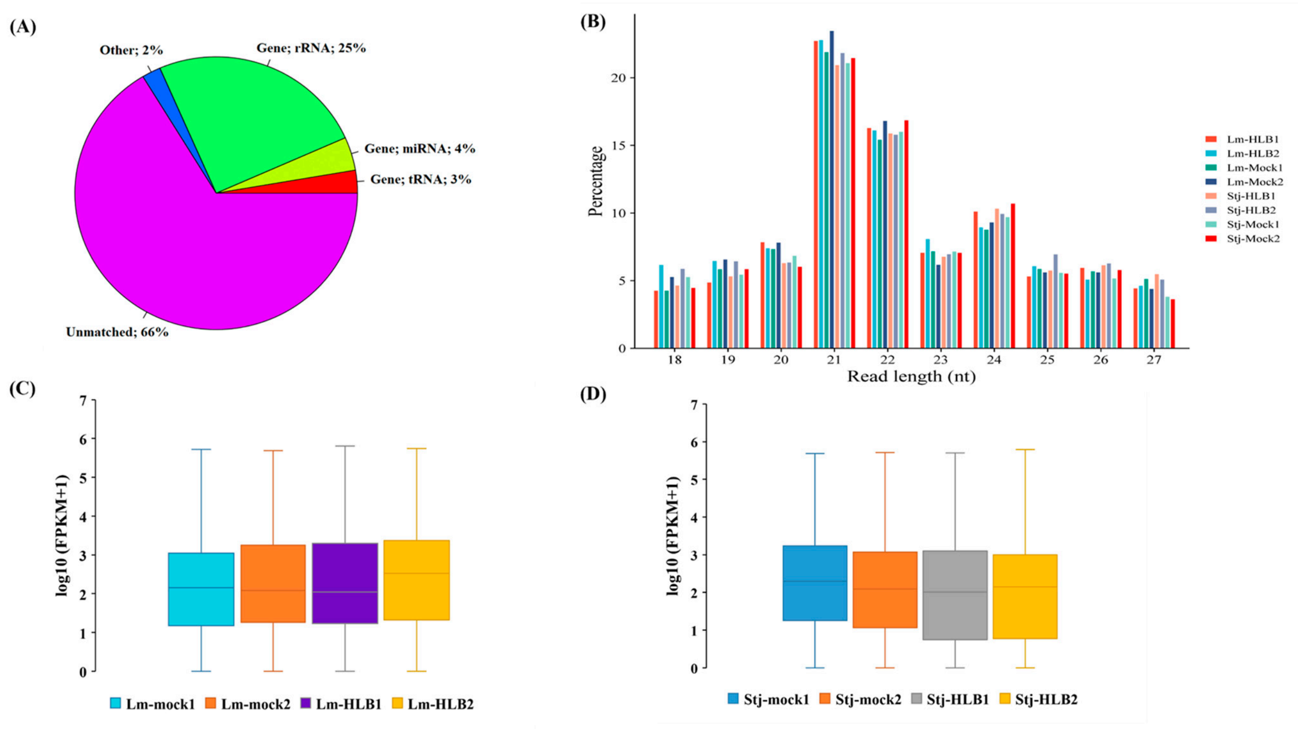

Figure S3: An overview of sRNAome profiling**.** (A) The pie chart elucidates different classes of RNA against Rfam (B) length distribution analysis of sRNAs in each library. The X-axis represents read length in nt, and the Y-axis shows the frequency percentage for each bar. (C-D) Boxplots show the distribution of known miRNA expression levels in each sample, and the dispersed degree of the data distribution can be observed. The X axis is the sample name and the Y axis is log10 (FPKM + 1). The boxplot of each area corresponds to five statistics (Maximum, upper quartile, median, lower quartile, minimum).

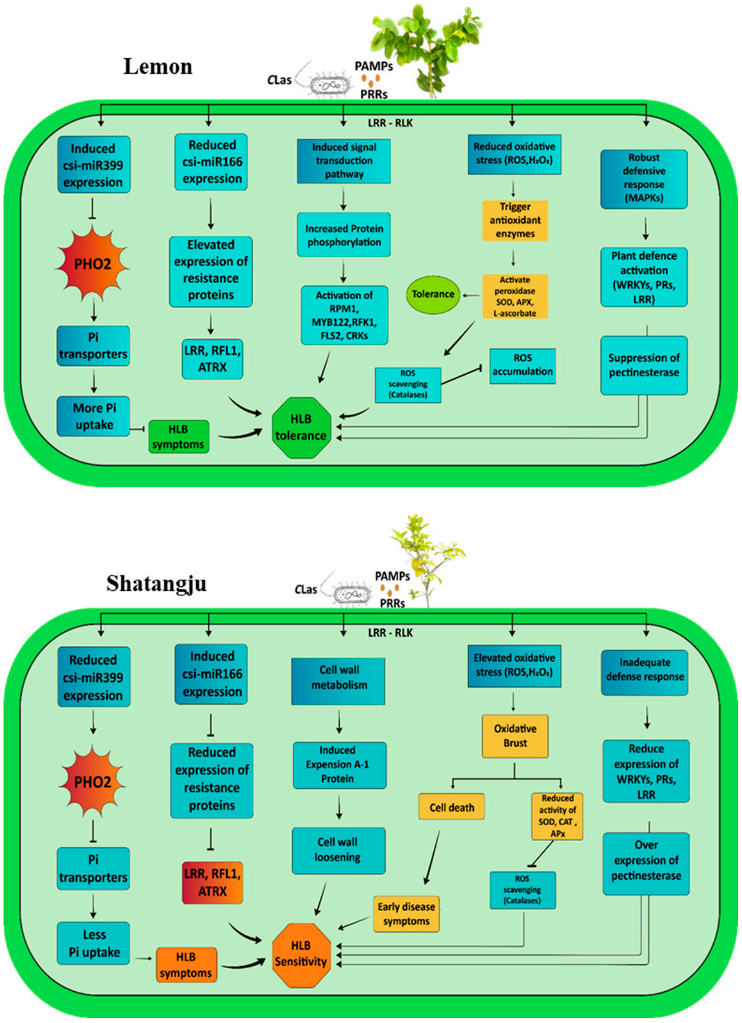

Figure S4: Schematic overview of significant pathways and DE-miRNAs of HLB-tolerant (*C. limon*) and HLB-susceptible cultivar (*C. reticulata* Blanco cv. Shatangju) upon CLas infection.

References:

1. Zhou, B.; Wang, J.; Guo, Z.; Tan, H.; Zhu, X. A simple colorimetric method for determination of hydrogen peroxide in plant tissues. *Plant growth regulation* **2006**, *49*, 113-118.
2. Oberley, L.W.; Spitz, D.R. [61] Assay of superoxide dismutase activity in tumor tissue. *Methods in enzymology* **1984**, *105*, 457-464.
3. Hodges, D.M.; DeLong, J.M.; Forney, C.F.; Prange, R.K. Improving the thiobarbituric acid-reactive-substances assay for estimating lipid peroxidation in plant tissues containing anthocyanin and other interfering compounds. *Planta* **1999**, *207*, 604-611.
4. Aebi, H. Catalase in vitro Methods Enzymol 105: 121–126. Find this article online. **1984**.
5. Asada; Kozi. Chloroplasts: Formation of active oxygen and its scavenging. *Methods in enzymology* **1984**, *105*, 422-429.
6. Boava, L.P.; Cristofani-Yaly, M.; Machado, M.A. Physiologic, anatomic, and gene expression changes in citrus sunki, poncirus trifoliata, and their hybrids after 'candidatus liberibacter asiaticus' infection. *Phytopathology* **2017**, *107*, 590-599.

7. DuBois, M.; Gilles, K.A.; Hamilton, J.K.; Rebers, P.A.t.; Smith, F. Colorimetric method for determination of sugars and related substances. *Analytical chemistry* **1956**, *28*, 350-356.
8. Livak, K.J.; Schmittgen, T.D. Analysis of relative gene expression data using real-time quantitative PCR and the 2− ΔΔCT method. *methods* **2001**, *25*, 402-408.

Disclaimer/Publisher's Note: The statements, opinions and data contained in all publications are solely those of the individual author(s) and contributor(s) and not of MDPI and/or the editor(s). MDPI and/or the editor(s) disclaim responsibility for any injury to people or property resulting from any ideas, methods, instructions or products referred to in the content.
